# No departure to "Pandora"? Using critical phenomenology to differentiate "naive" from "reflective" experience in psychiatry and psychosomatic medicine (A comment on Schwartz and Wiggins, 2010)

**DOI:** 10.1186/1747-5341-5-15

**Published:** 2010-10-31

**Authors:** Jann E Schlimme, Catharina Bonnemann, Aaron L Mishara

**Affiliations:** 1 Department of Philosophy, Karl Franzens University Graz, Graz, Austria; 2 Department of Psychiatry, Social Psychiatry and Psychotherapy, Hannover Medical School, Hannover, Germany; 3 Department of Clinical Psychology, Chicago School of Professional Psychology, Chicago (IL), USA

## Abstract

The mind-body problem lies at the heart of the clinical practice of both psychiatry and psychosomatic medicine. In their recent publication, Schwartz and Wiggins address the question of how to understand life as central to the mind-body problem. Drawing on their own use of the phenomenological method, we propose that the mind-body problem is not resolved by a general, evocative appeal to an all encompassing life-concept, but rather falters precisely at the insurmountable difference between "natural" and a "reflective" experience built into phenomenological method itself. Drawing on the works of phenomenologically oriented thinkers, we describe life as inherently "teleological" without collapsing life with our subjective perspective, or stepping over our epistemological limits. From the phenomenology it can be demonstrated that the hypothetical teleological qualities are a reflective reconstruction modelled on human behavioural structure.

## I. Introduction

In their recent paper Michael A. Schwartz and Osborne P. Wiggins [1] discuss the difficulty of integrating hermeneutical/teleological and scientific/biological approaches to understanding living beings. They point out that traditional solutions so far as they presuppose "metaphysical dualism" fail to integrate these perspectives due to their inability "to explain the *relationship *between mind and body [...] because it was obvious from our own daily experience that mind and body were intimately united." Moreover, they contend that our culture crossed a point of no return with Darwin's theory of evolution. According to Schwartz and Wiggins, we are no longer able to keep the two perspectives of our life separate. Nevertheless, we would like to point out that human cognitive development is characterized by a fundamental "common sense dualism" between mind and body [2-4]. This "common sense dualism" is therefore so built-in or accustomed, or as we like to say, "embodied" in a fundamental sense. As Hume argued, it prereflectively determines our reflective experiences: "Custom operates before we have time for reflexion." [[5], p. 72] Despite well-meaning efforts of researchers and philosophers to view the mind and brain as ultimately the same, the tendency towards a dualistic thinking is nearly intractable.

Moreover, this deeply embedded common-sense dualism is reflected in the blockbuster hits like "Matrix" [6] or online games like "World of Warcraft," albeit as expressed in terms of a virtual reality. These postmodern myths suggest that we are able to transform our experience of self as if it were merely a matter of changing the "software." That is, we seem to find ourselves in the midst of a prevailing cultural "hysteria" in response to the recent advances in neuroscientific research. The latter is often interpreted as inevitably leading to a *neurobiological determinism*, be it feared or revered as an ultimate solution, whether one chooses to side with its proponents or opponents [7]. The recent blockbuster "Avatar" goes even further. In "Departure to Pandora" [8], soulless, but living bodies are able to be "animated" through mind-travel by employing highly sophisticated technology. With the help of a (herbal) biological mega-network the Na'vi (indigenous to the planet Pandora and a kind of noble savage) make the bodiless mind-travel of a young marine (protagonist of the story) able to take up permanent residence in his avatar-body. "Pandora" therefore may express the cultural wish to no longer be burdened by the human reflective experience of a nearly intractable dual relation to our own bodies in nearly every sphere of experience.

We agree with Schwartz and Wiggins on the point that metaphysical dualism is not able to explain the incongruity between mind and body, but question whether they are correct when claiming that Darwin's theory of evolution marks what we call here a point of no return to "Pandora", a land of animism and metaphysical-commonsensical dualism?

## II. Phenomenology: Assuming a Methodically Critical Attitude

We raise the following question in this paper: Are the epistemological limits of consciousness the crucial point in defining this point of no return to metaphysical-commonsensical dualism? We shift the focus of argument to the following question: Why are we no longer able to hold onto our original commonsense dualism when approaching these topics scientifically? For example, many contemporary neuroscientists insist that phenomenal (mental) experiences (qualia) are materially identical to the underlying neural processes but then are unable to explain how this occurs. This is the well-known "explanatory gap" [9] or the "hard problem" of mind-body-duality. Our primary point here, and one that Schwartz and Wiggins have themselves often reiterated in their writings: whenever we make generalizations about human experience, i.e., propositional statements about the nature of human subjectivity, we should be able to recount in a methodological manner how we arrived at these conclusions. Husserl's phenomenological approach is helpful here because he sees phenomenology as critique in two ways [10]: 1) as critique of assumptions which uncritically inform theoretical models of human subjectivity; and 2) as "critique" (in the Kantian sense) in the sense of being able to recount the necessary conditions of possibility in human subjectivity for any knowledge claim to take place. Here Husserl, the founder of the phenomenological method, applies his phenomenological "reductions" (literally a leading-back from the original Latin, see below) including his genetic phenomenological approach, which uncovers the passive-automatic (pre-reflective) processes [see also [11-13]], which inform human cognition.

In the wake of Kant's critical philosophy, the celebrated German lyrical poet, Friedrich Hölderlin (1770-1843) examined how human reflection inevitably takes itself into account when approaching its own claims of knowledge with regard to pre-reflective human experience. According to the contemporary philosopher, D. Henrich [14], Hölderlin for the first time depicts in his philosophical fragment "Urtheil und Seyn" („Judgment and Being,“ 1794/95), "reflection as an active step towards a ground, which in itself is not knowledge but nevertheless serves as the immanent basis for this knowing" [[14], pp. 99-100, our translation] Similarly, the celebrated founder of psychosomatic medicine in Germany, Viktor von Weizsäcker [15] (see also below) describes subjectivity as a "fundamental relationship" (*Grundverhältnis*) to experience *which simultaneously does not know itself*. According to these thinkers, it is of crucial importance to remain aware of these limits especially when transferring concepts from their formulation in reflective or scientific contexts back to some kind of putative anchoring in our naïve unreflected experience.

Relying on the prior work of Dilthey and Weber, Jaspers [16] emphatically pointed to the differences between methods of the human sciences (*Geisteswissenschaften*) and those of the natural sciences. However, this methodic opposition, which is merely a nod to commonsensical mind-body dualism (or the so-called explanatory gap) is not satisfactory. We are convinced that it is important to adopt (and retain) a "methodically critical attitude" (in German, "methodenkritische Einstellung", see [17]), i.e., an attitude which is critically aware of the methods it employs and the inhering limits of these methods. Importantly, this critical attitude does not itself yield to either side in the debate between the human and natural sciences. In describing this attitude, we rely on Husserl's transcendental phenomenology. The latter is to be understood as a "critical attitude" rather than a "tool-box," which is able to produce manifest results that are comparable to, or on a par with the experimental findings of cognitive science/neuroscience. Despite Jaspers pointing to the methodic opposition between the human and natural sciences, this critical attitude is advocated by the later Jaspers [18]. We suggest that it is precisely this critical attitude that is fundamental to the phenomenological position.

The phenomenologic-critical methodic attitude allows us to track abstract constructs such as metaphysical-commonsensical dualism as somehow inserted in the difference between our 'natural-naive experience' and our reflection on this experience. Phenomenological reduction is a "leading back" (from the Latin *reducere*) from one's current engagement with the world to examine (reflectively) the "streaming-consciousness" in the here and now; this requires the bracketing of common sense folk-psychological/folk-physical assumptions about how minds and objects behave in the world. That is, in its step-wise method of reduction as a reflective stepping back from natural engagement with the world, imaginitively varying (eidetic reduction; *Wesensschau*), abstracting, attempting to verbally "fix" (Husserl) in descriptive language, phenomenology implements reflection, which is always retrospective to the experience it reflects on, as its sole instrument [19]. Following Husserl, making propositional statements about the nature of human subjectivity requires that we critically reflect on our own assumptions, thought processes and conclusions. This implies that we are not able to state metaphysical facts, but can only describe the structure of "how" our experiences and experienced things are given to us. We therefore introduce this methodical caveat as segway into our following discussion of the mind-body-problem, teleological life-qualities, and how these become available to the methodologic distinction between the "natural" and "reflective" experience.

Eccentric positionality and its impact for mental disturbances:

We remain agnostic whether reflection introduces the gap between mind and body or rather itself becomes possible because the split is already there pre-reflectively (for Husserl's view, see [19]). The phenomenological anthropologic thinker, H. Plessner argues that the human condition is characterized by an ambiguous relationship to one's own body, the fact that one can experience one's own body as both subject and object, which Plessner calls "eccentric" or "broken" positionality: "If the life of the animal is centric, then the life of the human, without able to break through this centric orientation, is, at the same time, outside it, eccentric..." [[20], pp. 291-292, our translation]. Eccentric positionality, "human being-in-the-world," is the paradoxical relationship to our own embodiment as being both embedded in situations and outside them, as external to the vital centric-viewpoint, experiencing ourselves from without [21]. That is, the ability to explicitly reflect on our experience and commonsensical mind-body dualism may emerge from the same source, human eccentric positionality, which is already preformed via the pre-reflective structure including our body and customs as can be especially seen in addictive behaviour [22]. Eccentric positionality is also the condition of human vulnerability to trauma [23] and psychosis [24,25].

## III. The "Mind-Body-Problem"

The importance of debating the mind-body-problem becomes clear when considering what Schwartz and Wiggins write about the phenomenon of "life" in their article. When trying to grasp "life", we find ourselves faced with the problem of "natural vs. reflective" experience. Schwartz and Wiggins [1] observe: "we can claim *privileged access: since we are living beings ourselves, we know what it means to be alive from our own first-hand experience*." In our opinion this privileged access does not change or solve the problem, but is the essential basis for having the problem.

After all, Schwartz and Wiggins' statement: "Every moment of our waking lives we directly experience life, life in ourselves and in others" is a truism. On the other hand, it is a claim about our pre-reflective experience that cannot be verified reflectively. To say that we "directly" experience our own living from moment to moment suggests that some kind of sentience of life must accompany each of our cognitions, a statement that requires verification. In his "Material Phenomenology," the French theist philosopher, Michel Henry [26] proposes that "[l]ife is a how, both a mode of revelation and revelation itself" (p. 119). Yet, we must be very careful here to guard against Henry's metaphysical conclusion (not based on the phenomenological method): that since we are alive we have *immediate *access to our own "how" as transcendental condition for this living. In his writings, Henry repeatedly makes the methodic mistake of confusing phenomenologic method for results. Henry [27] claims this presupposed immediate access to the "how" of our naïve pre-reflective experience as passive auto-affection. However, this "how" is only made available as a verbal-abstraction subsequent to phenomenological reduction and therefore heavily laden with reflection's own armour. Henry's position apparently requires that the reflective and the pre-reflective quality of experiences would be the same as if the reflecting subject could fold back on itself and somehow be the same in both the pre-reflective and reflective perspectives, despite the "delay" that the reflection itself requires.

As already stated, we are clearly alive, and in the instance of bringing this fact into our focus, we are retrospectively referring to our own experience. In doing so we are unable to bring the act of the hyperbolic-reflecting itself (being itself the focusing) into focus. Of course, this argument seems to be in danger of "infinite regress" and to call for fundamental scepticism. However, we do not agree with this point for the following reason. Having privileged access, we are inevitably alive in the fullest sense while experiencing this privileged access. *But even though being alive in the fullest sense, we are unable to obtain a "full picture" of this living itself, i.e., having "immediate" access to this life as a totality without some sort of cognitive, affective or interoceptive mediation*. After all, any reflective access to our own living as a totality must be a retrospective *pars pro toto *relation to this totality. Something is always left out within this act of reflection namely the antecedent, pre-reflective basis of conscious experience which is not available to this reflecting except as a "decaying" retention sinking into the, what Husserl calls, the "night of the unconscious" [28,11]. Even if I claim that I am always interoceptively aware of my own body in its inner milieu as the presupposed "background" of each conscious now, it is still pars pro toto. To put it simply: though the "natural experience" is given fully to the subjective perspective engrossed in this experience, it cannot be taken fully into account. Because our positionality is ultimately ec-centric (Plessner, see below), even with respect to our own embodied living, we may not so easily dismiss, or presume to solve the mind-body-problem [see also [29,30]]. It continues to be the "hard problem". The German philosophical anthropologist, Helmuth Plessner distinguished being a body as subject (Leib-sein) from having one's body as object (*Körper-haben*). Human subjectivity is *Leib im Körper*, that is, the body as subject (*Leib*) is lodged in but not coincident with our awareness of the "same" body as object (*Körper*): "The person *is *always subjectively lived body (*Leib*) (with head, trunk, appendages and all that such experiencing includes) - even if this same individual is convinced that his own immortal 'soul' is also present inside his *Leib *- and, at the same time, he *has *this body as corporeal object (*Körper*)." [[31], p. 43 our translation]. "My embodied being-in-the-world as self-transcendent is ec-static, prospectively open and vulnerable to the not-yet-known in a way that extends beyond my experience of having a 'self-enclosed body image'." [[32], p. 621]. The fundamentally human relationship to body is ambiguous in that we are able to shift frame of reference with regard to the "same body": we are able to take on ***both ***an internal-vital (i.e., proprioceptiveinteroceptive) and external (exteroceptive, social-objectifying) relationship to our own bodies. The latter is experienced as a self-enclosed entity in that I take an external or exteroceptive relationship to my own body as "object," anticipating its totality as others might experience/see it. Although the term "body ownership" has become quite fashionable in recent phenomenologic and neuroscientific writings, this is not quite correct. It is not merely that I "have" (or "own") this body or my body image (which is exteroceptively given to me, but also imagined as object). *It is not just "mine" (exclusively my province, accessible only to me as my own interoceptive experiences are) but is also how I imagine others to experience me*. As Plessner writes: "The visual presenting of my body to myself, always an external relationship, obtains a main role in conveying a total-impression to myself, but mainly of the frontal part of the body, and with the important exception of the head, and the region decisive for social contact [and reciprocity of perspectives], the face...With this visual incompleteness..., especially the invisibility of one's own face, we come to...the *image *of our own bodies (*Leibimago*)" [[33], vol. 8, pp. 292-3 (cited and trans by Mishara [21])].

It is possible that those who argue that the naively or naturally experiencing pre-reflective subject has direct sentience of its own living from moment to moment may want to base their argument on claiming that this persistent life-sentience is given *in the background *as a kind of interoception in each conscious experience. The latter would indicate that these experiences are infused with a sense of "mineness" as a kind of tacit "ownership" of the experiences in interoceptive pre-reflective self-affection. This point of view was recently proposed by E. Thompson [34]. There are three problems with this view: 1) how could we know in the sense of an explicit knowledge this without first reflecting on it and thus thematically produce the very interoceptive experience we claim to be there in the background before the reflecting? Such putative requisite prereflective life-sentience is not available to the reflective-phenomenological method of "reduction"; 2) Since the subject is already experiencing these experiences, why is there the additional requirement that these experiences be "tagged" as "mine," the so-called experience of "ownership," in what is an unnecessary, nonparsimonious redundacy? *Such tagging would be relevant rather to an episodic memory system after the fact which implicates a remembered, embodied and situated self apart from current experiencing*, e.g., Tulving's "time travel" [35], see below. This relevance can be especially seen when functions of the episodic memory are impaired, e.g. in frontotemporal lobar degeneration, were people do not realize the changes of their abilities due to impaired functions of "context-recall" and "reexperiencing" [36]; 3) The current evidence in neuroscience does not support the redundant view: so-called "inner" or interoceptive awareness of being alive is subserved by an interoceptive reward-emotional pathway (e.g., in the inner feeling of vitality), e.g., [32]. However, neuroimaging, single electrode and related studies reveal that the brain pathways subserving interoception are not tonically active in the background from moment to moment [37], and therefore, in their very intermittency, not a *sine qua non *(contra Thompson) for conscious experience. Moreover, such putative tonic activity as an ongoing tagging would not be parsimonious from the point of view of brain evolution which conserves its limited resources (i.e., excitosis and reuptake or recycling of vital but finitely available neurotransmitters, such as dopamine) to signal precisely those events which hold some significance for the organism or the organism's survival. That is, the interoceptive signalling (e.g., Damasio's so-called somatic markers) so far as it indicates important moments of "salience" (presumably relevant to the organism's survival or biologically meaningful in some other way) and thus, to be *noticed and learned *by the subject, is activated *phasically *(i.e., involving a "prediction error" between expected events and outcomes which are better or worse than expected which then activates dopaminergic and other signalling) [38].

There is the additional problem, which we call here, a "soft" problem (to distinguish it from the "hard problem", or mind-body-duality, and the related "explanatory gap" between phenomenal-mental experience (qualia) and neural process we have been discussing up till now): even if we perform "reduction" in a Husserlian sense, we are never totally free in our reflection from historical consciousness, and from language which makes such methodic reflective "reduction possible." H.-G. Gadamer [39] called this inevitable historical consciousness the pre-reflective precondition of understanding (*präreflexive Vorstruktur des Verstehens*). Moreover, we are not able (contra Schwartz & Wiggins) to "start from both sides - from the side of what empirical science can tell us about inorganic and organic reality and from the side of our own direct experience of life in ourselves and in others" [1]. These perspectives are not only not the same, they methodically exclude one another in what Viktor von Weizsäcker [15] calls a Gestalt-circle (*Gestaltkreis*). Their difference depends on the respective way, or applied method which brings "things" into focus. With respect to the phenomenon of "life", it is not at all clear whether these different, mutually exclusive approaches focus on the same phenomenon. Obviously there is a difference between observing living cells through a microscope and understanding Homer's concept of life. At any rate, we acknowledge that certain characteristics may be "physiognomically" given when experiencing something which is alive, or saying something about the structure of our experience as a living being. Viktor von Weizsäcker, who, as we already noted, is regarded as the "founder" of psychosomatic medicine in Germany, had argued that we must introduce the concept of subjectivity into the study of life or biology. However, *as reflecting subjectivity, we do not have access to the "hidden unity" between mind and body in what von Weizsäcker calls the fundamental relationship (Grundverhältnis) to our own being*. Mind and body are, as can be said with reference to Aristotle, rather experienced as "two sides of the same coin" [40,41]). That is, mind and body are in "hidden" unity or *Gestaltkreis*, which is not given directly to consciousness whether in pre-reflective naïve experiencing, or the reflection on this experiencing. In our view, it is only through acknowledging this human finitude (rather than claiming that "mind-body dualism" has been overcome) that a psychosomatic medicine or a philosophically informed psychiatry can be properly practiced.

## IV. Teleological Qualities of Life

If we accept the formerly described aporia of trying to resolve reflective and pre-reflective, or "reflective" and "natural" experience, we come to the conclusion that the "teleological quality of life" depends on a self-reflective act. Of course, we prereflectively experience our behaviour as goal-directed; there is "telos" in all behaviour in this sense, even in the most accustomed or habitually motivated behaviours. However, this is not equivalent with "teleology" which always implies explicit teleological interpretation. A rolling stone does not roll as it were on teleological accounts. Rather, so-called teleological qualities are only given when (self)-conscious beings interpret their own experiences in a broader behavioural context. This does not diminish the quality of teleological interpretations, but it clarifies their status. Notably when Husserl describes a drive-intentionality (*Triebintentionalität*) as fundamental to the passive originary association (Urassoziation) of each "living" present or current moment of consciousness, he means that we proleptically strive for completeness of the meaning of each experienced event as it passes in time consciousness (see also [15]). *However, even here, the teleology is provisional, an anticipated totality of meaning, which is always reliant on further perspectival refinements or elaborations of the original experiencing*. In a similar manner, Kant instructs us about our epistemological limits. According to Kant, it is not possible to decide the overall or all encompassing ("metaphysical") sense and meaning of our being alive. It is also impossible for us to decide whether - despite its obvious importance for ethical decision-making (i.e., a metaphysics of morals) - there is such a "metaphysical" sense at all (in the so-called "teleological limits" of the regulative idea). In his "Kant-Crisis" of 1801, the German writer and novelist, Heinrich von Kleist (1777-1811) [42-44], experienced this insight as highly vexing to his own sense of existence. Later in the century, Arthur Schopenhauer (1788-1860) described the foreboding consequences when the meaning of existence remains non-securable in a "metaphysical" sense: "To what end the entire tragic-comedy (of our lives) occurs is not to be determined even remotely. Moreover, there is no audience, and the actors of life simply just hold out in what seems to be endless toil, with little, or a merely negative pleasure." [[45], Bd.3, pp. 416-7, our translation]. As argued above, these limitations are in a radical sense dependent on the "hard problem", which we have reformulated as the difference between our "reflective" and our "natural" experience (and the attendant problem of mind-body-duality). However, we must be careful that such irritative-affective accounts of biologic subjectivity do not overlook its very structure as enabling hidden unity with itself and its environment as von Weizsäcker, pointed out [15,32]. The latter is not possible by collapsing the dualism - by jumping over the explanatory gap - but through acknowledging the relationship of mind and body as one that intrinsically (but concealedly) connects them.

The knowledge of the limits to our own metaphysical reflections leads to the awareness that the experienced meaning of our life is deeply intertwined with the way we live. This was painfully obvious to the existentialists who argued that we ultimately take responsibility for the meaning of our own lives. We therefore agree with a modified version of Schwartz' and Wiggins' thesis. Rather than claiming as they do that "Life is thus *teleological*: the present activity of the living being *aims at *its own future being", we offer the following: "Life seems teleological for subjects when they are in the situation of aiming at anticipating their own future in their present activity, and at the same time, are reflectively aware of their presumably pre-reflective and accustomed aiming." If we refine their conclusion in that way, we acknowledge that people *experience *a teleological quality in their embodied life-situation. But this teleological quality is only grasped as it is consciously given. To experience it affords its active "reconstruction" in a subsequent reflective act, while all the time referring to one's "natural experience," the putative pre-reflective experienced directedness of one's behaviour, on which the teleological interpretation is modelled in a structural sense. In other words, the experienced teleological quality of life can only be understood as an interpretation with constant and undeniable reference to this experienced behavioural structure or actional structure ("*Handlungsstruktur"*, see also [46]).

## V. Discussion

Schwartz' and Wiggins' [1] suggestion that the phenomenon of life is given in a polarized way is attractive and helpful and certainly has its precedents (e.g., Plessner, von Weizsäcker). For example, von Weizsäcker [15] writes: "Death is not opposite to life, but rather a counter-player or antagonist to procreation and birth; birth and death rather comport themselves like the back and front sides of life, and not as logically mutually exclusive opposites. Life *is *birth and death" (p. v, our translation). Admittedly, we are not be able to "introduce value into an otherwise value-free universe" in the sense that we could freely invent values without them being defined by our "natural experience" (contra Sartre, but in consilience with Scheler). It is nevertheless a reflective act in which an experienced givenness (e.g. a certain circumstance) is *named *as a given value. Reflection allows us to describe our own experience of being alive in polarizing qualities as inside and outside, being embodied/embedded vs. being disembodied and sovereign, sameness and processual change, self and other. Yet the distinction between pre-reflective and reflective lived experience is surely one of the most polarizing and most vexing!

For example, it is possible to interpret the life of a neuron as it finds its way to appropriate targets in migratory neurogenesis, for example, to be "teleological" in our human sense. However, this may merely be in psychoanalytic terms the "projection" of animistic belief. Although clearly unable to say anything without such neural activity, we nevertheless are also unable to state what it is like to be this nerve cell. We remain critical when speculating on the teleologic character of life, organisms or processes outside the experience of goal-directedness of our own human consciousness. For example, Schwartz and Wiggins [1]: "The being that the activity is geared toward preserving is the organism's future being... Life is thus teleological." (p. 3). But is it really a "future" life that the organism is geared toward preserving from moment to moment, or rather, *is it only its current being-alive which the animal strives to preserve but which displaces itself which each new "now" into what we, as observers, call a "future." *Although the animal procreates and often cares for its young, the ethical concern of preserving life and the environment for future generations is, as Jonas [47] himself argues, an exclusively human responsibility (as is, vice versa, the possibility to end one's life on one's own explicit account). The human affective-openness to world (*Weltoffenheit*), the vulnerability to a future which is not yet, i.e., anticipatory affective alterity as subjectively "felt," is for the phenomenologic anthropologists (e.g., Binswanger, Plessner, von Weizsäcker), something we can only attribute to human existence (not something we can speculatively attribute to "animal minds"). Moreover, "meaning" and the human task of finding meaning in existence is never coincident with life (as can be seen in the well-known fact that we, as human beings, are able to utterly despair, as Kierkegaard argued [[48], p. 9ff.]). It is always an excess, an overshooting, precisely because it always undershoots, never quite captures the living that "underlies" it as its hidden source. Similarly, Tulving and other cognitive psychologists attribute mental "time travel," the ability to have explicit retrospective and prospective episodic memories, and thus an explicit future, and its underlying neurocircuitry, to a specifically *human *awareness [see [23]; though of course, this may not be the final word on this topic].

As Schwartz and Wiggins point out, those who claim that we are able to make such "teleological" accounts draw on a metaphysical dualism. They animate things of the world as if those were self-conscious in a human way, yet "forgetting" at the same time that their belief is an interpretation which depends on their preferred way of accessing their world experience or world-view (in Dilthey's sense).

Such approaches may attempt to justify a bodiless soul which, from the standpoint of thought alone is possible to conceptualize as a metaphysical entity but is not justified according to phenomenology's own critical method [49]. The relationship to the presumed givenness of self-evidence in delusions may have a similar structure [50,51]. From this point of view, it is not Darwins's evolutionary theory that keeps us from departing to "Pandora", but it is the (phenomenological) methodically-conscious attitude ("methodenkritische Einstellung"). As a child of enlightenment, Darwin also advocates methodically critical thinking. We propose that the difference between "natural experience" and "reflective experience," and how this difference still contributes to the hard problem, should serve as antidote for those who search futilely for "Pandora." We should remind them, and we may also thank Schwartz and Wiggins' recent and thoughtful article for this reminder, of human finitude and our own epistemological limits.

## Declaration of competing interests

The authors declare that they have no competing interests.

## Authors' contributions

JS, CB and AM conceptualized, drafted and revised the manuscript and contributed equally to its development. All three authors read and approved the final manuscript.
